# IL-10/STAT5 axis suppresses miR-140 to upregulate B7-H4 expression in RAW264.7 cells

**DOI:** 10.3389/fcimb.2025.1613297

**Published:** 2025-08-15

**Authors:** Dandan Zhu, Guo Chen, Pei Shen, Weiliang Fan, Chuxin Ji, Yinong Duan, Wenxi Gao

**Affiliations:** ^1^ Department of Pathogen Biology, School of Medicine, Nantong University, Nantong, Jiangsu, China; ^2^ Department of Dermatology, Huashan Hospital, Fudan University, Shanghai, China; ^3^ Department of Laboratory Medicine, Affiliated Hospital of Nantong University, Nantong, China; ^4^ Laboratory Center, School of Educational Sciences, Nantong University, Nantong, Jiangsu, China

**Keywords:** miR-140, B7-H4, IL-10, STAT5, *Schistosoma japonicum*

## Abstract

**Introduction:**

*Schistosomiasis japonica*, a zoonotic parasitic disease, induces complex immune regulation during infection. The inflammatory responses and immunosuppressive mechanisms co-exist to maintain immune homeostasis in schistosomiasis. B7-H4 is a critical immune checkpoint molecule that modulates T cell activation and exerts immunosuppressive effects. Our previous investigations revealed that B7-H4 mRNA expression was elevated in mice infected with *Schistosoma japonicum*, with interleukin-10 (IL-10) demonstrating regulatory capacity to enhance B7-H4 expression in RAW264.7 macrophages. In this study, we further explore the mechanism underlying IL-10-mediated B7-H4 upregulation.

**Methods:**

Western blot was performed to detect B7-H4 expression levels, both in mice infected with *Schistosoma japonicum* and in RAW264.7 cells stimulated with IL-10. RT-qPCR was performed to screen microRNAs (miR-140 et al.) in RAW264.7 cells stimulated with IL-10. Then dual-luciferase reporter assay was performed to confirm that miR-140 can directly bind to the 3’UTR of B7-H4. miR-140 promoter activity in RAW264.7 cells was also detected via dual-luciferase reporter assays. In addition, ChIP was performed to confirm the binding of transcription factors and miR-140 promoter.

**Results:**

Notably, miR-140 was decreased in IL-10-treated microphages, accompanied by B7-H4 expression was upregulated. miR-140 can directly bind to the 3’UTR of B7-H4 and then inhibit the expression of B7-H4 in RAW264.7 cells. Meanwhile, miR-140 mimics can also attenuate IL-10-induced B7-H4 expression in RAW264.7 cells. Then we found that IL-10 may inhibit miR-140 promoter activity in RAW264.7 cells through transcription factors that binding to the - 576/- 94 bp region of the miR-140 promoter. Results by Western blot and ChIP further indicated that IL-10 could downregulate miR-140 promoter activity in a STAT5 dependence manner. After the sequence of STAT5 binding site within the - 456/- 446 bp region of the miR-140 promoter was mutated, IL-10 failed to suppress the activity produced by mutant miR-140 promoter.

**Discussion:**

In summary, IL-10 can inhibit miR-140 through STAT5, thereby upregulating the expression of B7-H4 in RAW264.7 cells. This study may suggest a new mechanism underlying IL-10-mediated B7-H4 upregulation in macrophages.

## 1 Introduction

Schistosomiasis is a parasitic disease caused by infection with schistosome (Ullah et al., 2022). After schistosome infection, schistosome eggs-induced granuloma formation and tissue fibrosis resulting from host immune responses are the main causes of high mortality in patients with schistosomiasis (Llanwarne and Helmby, 2021). In the early stage of schistosome infection, Th1 response is predominant and IFN-γ and TNF-α levels are increased. Then macrophages is activated to clear pathogens and Th2 response takes the dominant status gradually, allowing infection to enter the chronic phase (McRae et al., 2015).

The B7 family is the second signal of T cell activation and also a group of immune checkpoints, which are usually expressed in different immune cells, such as antigen presenting cells, T cells, B cells, and natural killer cells, and they play a crucial role in immune responses (Yu et al., 2024). As one of the important members of B7 family, PD-1 plays a critical role in inhibiting T cell function, and blocking of PD-1 blockade preferentially could enhance Th2 cell responses and ultimately lead to more severe liver immunopathological damage in mice infected with *Schistosoma japonicum* (Keir et al., 2008; Zhou et al., 2016). B7-H4 (also known as V-set domain containing T cell activation inhibitor 1, VTCN1) is another immune checkpoint that activates T cells and exerts immunosuppressive effects (Kryczek et al., 2006; Lin et al., 2021). As a co-inhibitory molecule of the B7 family, B7-H4 suppresses T cell receptor (TCR)-mediated proliferation, cytokine production and cell cycle progression by inhibiting Akt and ERK phosphorylation (Ni and Dong, 2017). In tumors, B7-H4 is high expressed, especially in cervical cancers, lung cancers or ovarian cancers. Its expression levels are associated with poor prognosis of these tumors (Kryczek et al., 2007; Han et al., 2018). Dehydroepiandrosterone, which is derived from adrenal cortex, exerts the antitumor function in the mouse xenograft models with oral squamous cell carcinoma. Meanwhile, dehydroepiandrosterone inhibits B7-H4 expression in oral squamous cell carcinoma cells (Wang et al., 2024). Conversely, in some autoimmune diseases, downregulation of B7-H4 exacerbates aberrant T-cell activation (Vaishnav et al., 2022). B7-H4 Ig protein also inhibits the proliferation of activated T cells in patients with type 1 diabetes and arrests cell cycle of T cells in G0/G1 phase (Ou et al., 2006).

Previous studies have shown that B7-H4 expression was upregulated in ovarian tumors and tumor environmental IL-10 could stimulate B7-H4 expression in macrophages (Kryczek et al., 2007, Kryczek et al., 2006). Importantly, IL-10 and IL-6 can be produced from tumor macrophages spontaneously, while B7-H4 was also produced from macrophages in an autocrine manner in human ovarian cancer (Kryczek et al., 2007). In cervical cancer tissues, there was a positive correlation between the expression of B7-H4 and IL-10 (Han et al., 2018). Given that *Schistosoma japonicum* infection induces both Th1/Th2 polarization and IL-10-dependent immunosuppression (Zhu et al., 2014), we hypothesized that B7-H4 may serve as a critical checkpoint to balance immune clearance and pathology. In our previous studies, we also found that B7-H4 mRNA expression was upregulated in mice infected with *Schistosoma japonicum* and IL-10 could promote B7-H4 expression in RAW264.7 cells (Data not published). However, the mechanisms by which IL-10 can induce B7-H4 expression in RAW264.7 cells have not been reported.

In this study, we further confirmed that B7-H4 protein expression was upregulated in livers from mice infected with *Schistosoma japonicum*. Since IL-10 indeed contributed the high expression of B7-H4 in RAW264.7 cells, we also further screened the microRNAs expression which may target B7-H4 expression and regulate the expression of B7-H4 in RAW264.7 cells.

## 2 Materials and methods

### 2.1 Mice experiments and ethics statement

Female C57BL/6 mice aged 6–8 weeks (n=18) were purchased from the Animal Center of Nantong University for schistosome cercariae infection. Briefly, the mice were randomly divided into two groups and named as infected mice (n=9) and uninfected mice (n=9). The infected mice were infected with schistosome cercariae (15 ± 2 per mouse) from the infected snails (National Institute of Parasitic Diseases, Chinese Centre for Disease Control and Prevention) via abdominal skin exposure. At 12 weeks after infection, mouse liver tissues were obtained and egg granulomas could be observed in infected mice. The liver tissues from uninfected mice were used as control.

All animal experimental procedures and protocols were approved by the Animal Committee of Nantong University (No 20200304-011) and efforts were made to minimize animal suffering.

### 2.2 Cell culture and treatment

RAW264.7 cells, a kind of mouse leukemia monocyte/macrophage cell line purchased from the Cell Bank of the Chinese Academy of Sciences (Shanghai, China), were cultured in Dulbecco’s modified Eagle’s medium (DMEM, Gibco, USA) containing 10% fetal bovine serum (FBS, Sciencell, USA) in a CO_2_ incubator at 37°C. For cell treatment, RAW264.7 cells seeded in 6-well plates were stimulated with IL-10 (10 ng/mL) for 24 hours or 48 hours.

### 2.3 Plasmid construction

Using Targetscan (http://www.TargetScan.org/) platform and the National Center for Biotechnology Information (NCBI, http://www.NCBI.nlm.nih.gov) platform, the binding sites containing AACCAC in B7-H4 3’UTR for binding to miR-140, and the miR-140 promoter sequence (−2000 bp to +201 bp) were both predicted. To construct B7-H4 3’UTR-related plasmid and miR-140 promoter-related plasmid, polymerase chain reaction (PCR) primers were designed (**Supplementary Table 1**). Then genomic DNA was extracted from RAW264.7 cells using the QIAamp^®^ DNA Micro kit (Qiagen, Germany). B7-H4 wild-type 3’UTR sequences or B7-H4 mutated-type 3’UTR sequences were inserted into the psiCHECK2 luciferase vector (Promega, USA) to construct B7-H4 3’UTR plasmids. The miR-140 promoter associated sequences with full length sequence, or with two truncated fragments and STAT5 binding site mutants, were cloned into the pGL3 enhancer vector (Promega, USA) to construct miR-140 promoter, miR-140 promoter A, miR-140 promoter B and miR-140 promoter mutant, respectively.

### 2.4 Chromatin immunoprecipitation

ChIP was performed by using the Simple ChIP kit (Cell Signaling Technology, USA). As the results by bioinformatics analysis (PROMO web online database), STAT5 binding sites existed in miR-140 promoter (− 456/− 446 bp region). Two pairs of primers for binding sites are designed as those shown in **Supplementary Table 2**. After RAW264.7 cells fixed by formaldehyde at a final concentration of 1%, chromatin was collected by sonication and enzymatic digestion. Anti-STAT5 antibody (Cell Signaling Technology, USA, **Supplementary Table 3**) was then used to pull down the DNA and IgG antibody provided by the kit was served as negative control. Then the purified DNA obtained via Simple ChIP kit was used as template and PCR was performed using two pairs of primers in **Supplementary Table 2**.

### 2.5 Western blot

Proteins were extracted using conventional methods. Briefly, tissue samples were homogenized on ice using RIPA buffer (Beyotime, China) and cell samples were treated with RIPA buffer by ultrasonic disruption on ice. After centrifugated for 15 min at 4°C, supernatants were collected and quantified using a BCA assay kit from Beyotime (China). Then the protein samples were separated in sodium dodecyl sulfate-polyacrylamide gel electrophoresis (SDS-PAGE) and transferred into PVDF membranes (Merck, Germany). Then the samples were incubated with 5% skimmed milk for 1 h and primary antibodies were applied overnight in a refrigerator at 4°C, followed by secondary antibodies for 1 h. Target proteins were detected using a chemiluminescence (ECL) kit (Merck, Germany). Density analysis was performed using Image Lab software from BIO-RAD (USA). Antibodies used in this study were provided in **Supplementary Table 3**.

### 2.6 Real-time quantitative PCR

MiRNAs were extracted from RAW264.7 cells using RNAiso for small RNA (TAKARA, Japan), and its concentration was detected in Ultra Microvolume Spectrophotometer (Nanjing Wuyi Technology, China). Then the RNA samples were reverse transcribed into cDNA using Mir-X miRNA First-Strand Synthesis kit (TAKARA, Japan). RT-qPCR was performed using SYBR Premix Ex Taq RT-PCR kit (TAKARA, Japan) in StepOnePlus real-time PCR system (Applied Biosystems, USA). PCR primers were provided in **Supplementary Table 4**, while the reaction conditions were provided in **Supplementary Table 5**. Relative expression levels of miRNAs were normalized to U6. The data were obtained and calculated using 2^-ΔΔct^ method by StepOne software (Applied Biosystems, USA).

### 2.7 Luciferase reporter assay

To assess the effect of miR-140 on B7-H4 3’UTR activity, mimics or inhibitors of miR-140, as well as psiCHECK2 luciferase reporter plasmids containing wild or mutant B7-H4 3’UTR were transfected into RAW264.7 cells using FuGENE HD Transfection Reagent (Promega, USA). To assess miR-140 promoter activity, miR-140 promoter, miR-140 promoter A, miR-140 promoter B, miR-140 promoter mutant were transfected into RAW264.7 cells, respectively. After transfection for 6 h, the medium was changed and then the cells were stimulated with IL-10. Luciferase activity was measured at 36 h post-transfection using the Dual-Luciferase Reporter Assay System (Promega, USA).

### 2.8 Statistical analysis

Statistical analysis (One-way ANOVA statistical analysis (LSD) or Independent-samples T test) were performed using SPSS 20.0 software and all data were obtained from at least three independent experiments and presented as mean ± SEM. P < 0.05 was considered as statistically significant.

## 3 Results

### 3.1 B7-H4 expression is upregulated and miR-140 is decreased in IL-10 treated RAW264.7 cells

**Figure 1:**
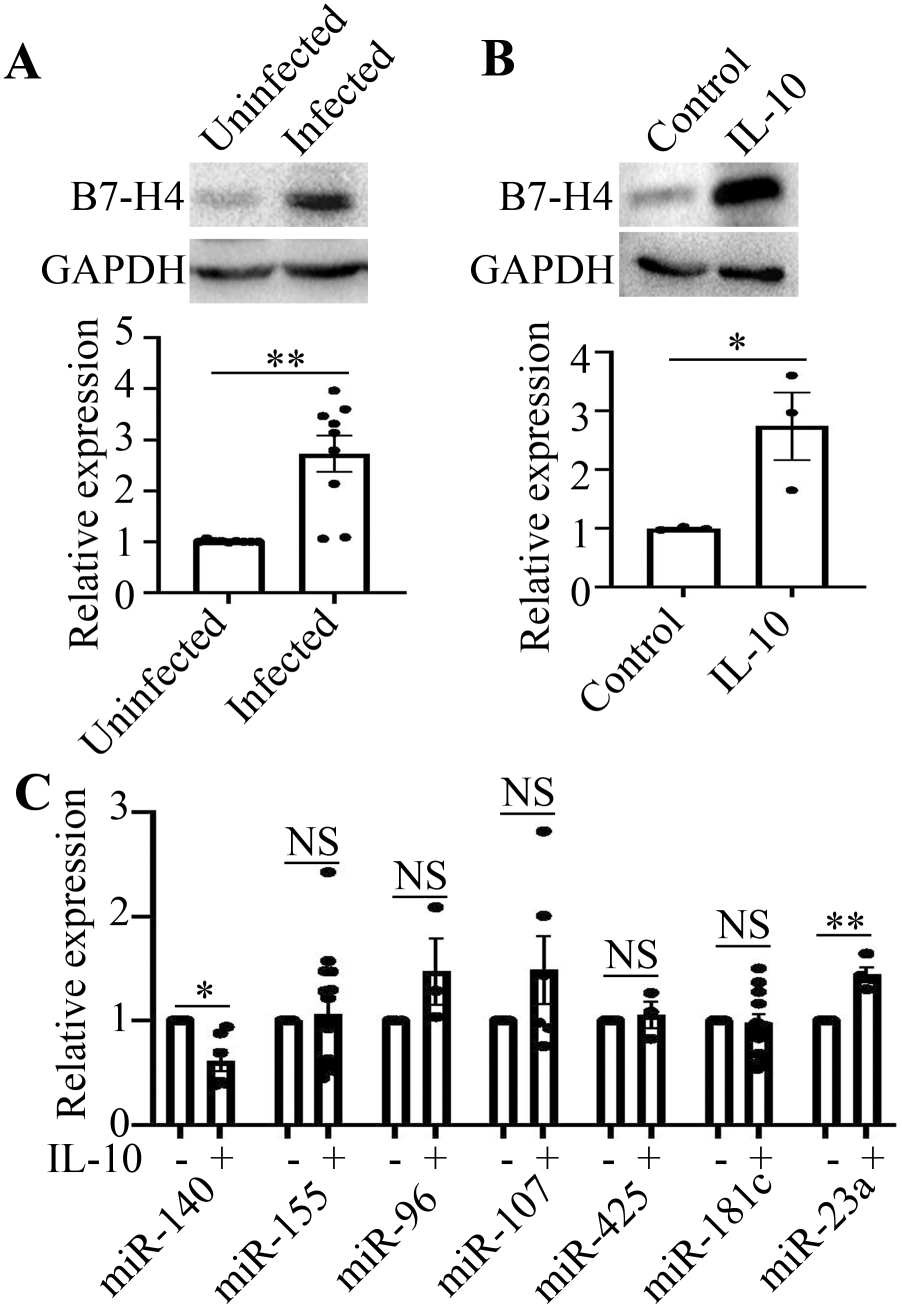
B7-H4 expression is upregulated and miR-140 is decreased in IL-10-treated RAW264.7 cells. **(A)**, The expression of B7-H4 in livers from mice infected with *Schistosoma japonicum* (n=9) was obviously higher than that from uninfected mice (n=9). B7-H4 expression was upregulated in mice infected with *Schistosoma japonicum*. **(B)**, The expression of B7-H4 in RAW264.7 cells stimulated with IL-10 was obviously enhanced, compared to that in RAW264.7 cells without IL-10 treatment. B7-H4 expression was upregulated in RAW264.7 cells stimulated with IL-10. **(C)**, miR-140 is decreased in IL-10-treated RAW264.7 cells, while miR-23a is increased in IL-10-treated RAW264.7 cells. No significant difference on other microRNAs expression (miR-155, miR-96, miR-107, miR-425, miR-181c) can be found between IL-10-treated group and control group without IL-10 (^NS^P>0.05). The comparison between the two groups was conducted using Independent-samples T test. *P<0.05 and **P<0.01 were considered as significant difference.

Firstly, we confirmed that B7-H4 protein expression was upregulated in livers from mice infected with *Schistosoma japonicum* for 12 weeks (**P<0.01, **Figure 1A**). Then RAW264.7 macrophages were stimulated with IL-10 (10 ng/mL) for 48 h *in vitro* and B7-H4 expression was detected by western blot. The results showed that the expression of B7-H4 protein was also upregulated in RAW264.7 cells stimulated with IL-10 (*P<0.05, **Figure 1B**).

By Targetscan database, we predicted that miR-155, miR-96, miR-107, miR-425, miR-181c, miR-23a and miR-140 may bind to 3’UTR of B7-H4 and regulate the expression of B7-H4. Therefore, the expression of microRNAs in RAW264.7 cells stimulated by IL-10 was detected by RT-qPCR. The results showed that the expression of miR-140 was down-regulated, while the expression of miR-23a was upregulated in IL-10-stimulated RAW264.7 cells (*P<0.05, **P<0.01, **Figure 1C**). IL-10 has no effect on the expression of miR-155, miR-96, miR-107, miR-425 and miR-181c in RAW264.7 cells. These results above demonstrate that B7-H4 expression is upregulated and miR-140 is decreased in IL-10-treated RAW264.7 cells.

### 3.2 B7-H4 is a potential target gene of miR-140

**Figure 2:**
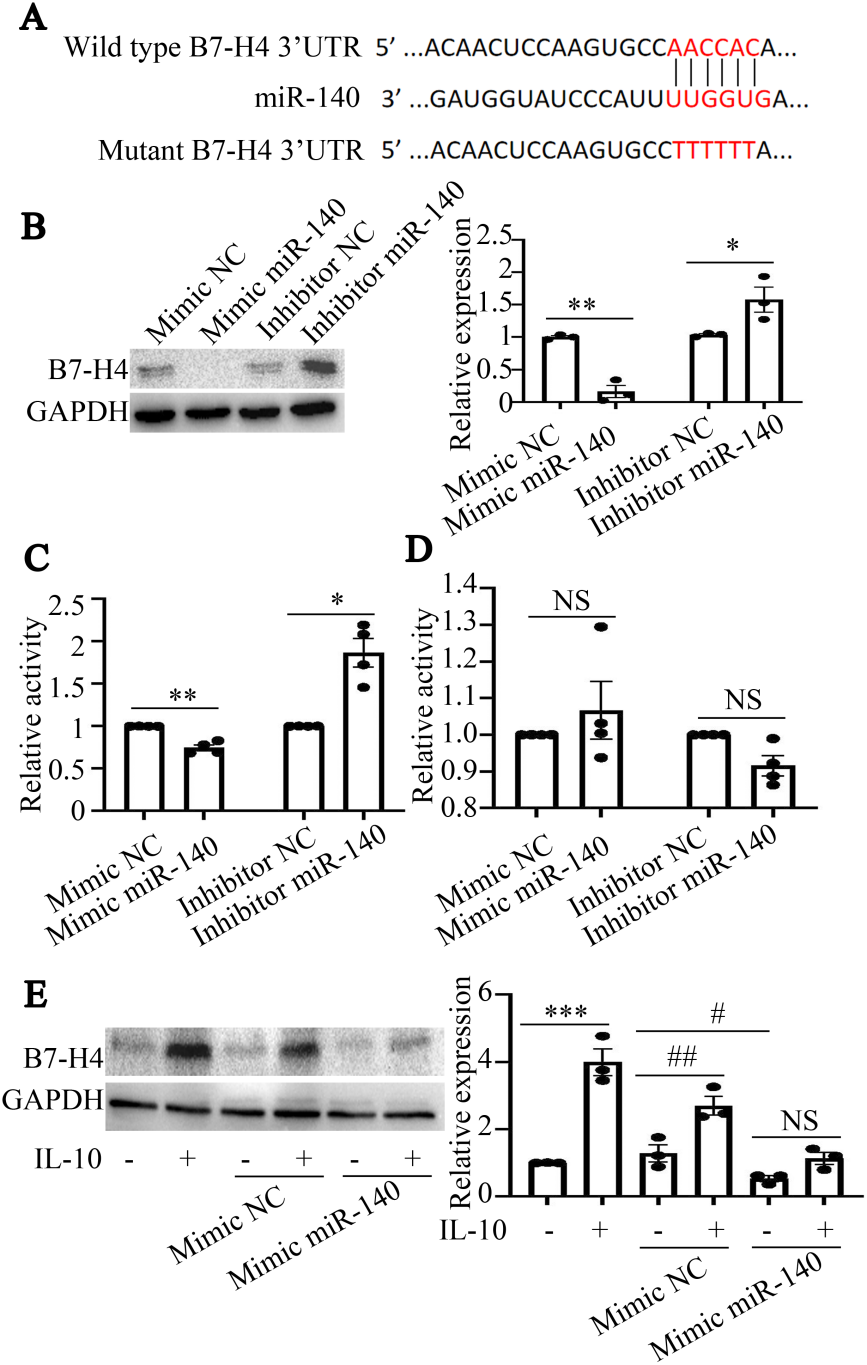
B7-H4 is a potential target gene of miR-140. **(A)**, The sequences of miR-140 and wild type or mutant B7-H4 3’UTR were shown. **(B)**, The expression of B7-H4 protein was detected by western blot. miR-140 mimics could inhibit the expression of B7-H4, while miR-140 inhibitors could promote the expression of B7-H4 protein. The comparison between the two groups was conducted using Independent-samples T test. *P < 0.05, **P<0.01, compared to each NC group. **(C, D)**, The fluorescence activities in cells transfected with wild type miR-140 3’UTR or mutant miR-140 3’UTR plasmids were detected by Dual-Luciferase Reporter Assay System. miR-140 mimics or miR-140 inhibitors could regulate the fluorescence activities produced by wild type miR-140 3’UTR transfection. miR-140 mimics or miR-140 inhibitors have no effect on the fluorescence activities produced by mutant miR-140 3’UTR transfection. The comparison between the two groups was conducted using Independent-samples T test. *P < 0.05, **P<0.01, compared to each NC group. **(E)**, B7-H4 protein expression was detected by western blot. In NC mimics transfected RAW264.7 cells or in no transfection cells, IL-10 could upregulate B7-H4 expression in RAW264.7 cells (***P<0.001, compared to mimics-IL-10- group; ^##^P<0.01 and ^#^P<0.05, compared to NC mimics+IL-10- group). After miR-140 mimics were transfected into RAW264.7 cells, IL-10 couldn’t upregulate B7-H4 expression in RAW264.7 cells (^NS^P>0.05). One-way ANOVA statistical analysis (LSD) was used to compare B7-H4 expression levels among the six groups.

According to predictions from online databases, B7-H4 may be a potential target of miR-140 (**Figure 2A**). To further explore the correlation between miR-140 and B7-H4 expression in RAW264.7 cells, mimics or inhibitors of miR-140 were transfected into RAW264.7 cells. As the results shown in **Figure 2B**, miR-140 mimics could inhibit the expression of B7-H4 protein in RAW264.7 cells (**P<0.01). However, B7-H4 expression was increased in miR-140 inhibitors transfected group, which was statistically significant compared to the inhibitor NC group (*P < 0.05, **Figure 2B**).

To determine whether miR-140 directly targets the B7-H4 3’UTR, miR-140 mimics or inhibitors, as well as wild-type plasmids or mutant plasmids of the miR-140 3’UTR, were transfected into RAW264.7 cells, respectively. Dual-luciferase results in **Figure 2C** showed that the fluorescence activity in cells transfected with wild-type miR-140 3’UTR plasmids was decreased by miR-140 mimics, while the fluorescence activity in cells transfected with wild-type miR-140 3’UTR plasmids was enhanced by miR-140 inhibitors (*P < 0.05, **P<0.01, compared to each NC group). However, in RAW264.7 cells transfected with mutant miR-140 3’UTR plasmids, miR-140 mimics or inhibitors couldn’t affect the fluorescence activity of mutant miR-140 3’UTR (^NS^P>0.05, **Figure 2D**). The above results indicate that miR-140 can directly bind to the B7-H4 3’UTR and then inhibit the expression of B7-H4 in RAW264.7 cells.

We further observed the effect of miR-140 mimics on B7-H4 expression in RAW264.7 cells treated with IL-10. As the results shown in **Figure 2E**, IL-10 could upregulate the protein expression of B7-H4 in RAW264.7 cells (***P < 0.001), while miR-140 mimics could inhibit B7-H4 expression in RAW264.7 cells (^#^P < 0.05). Importantly, in NC mimics transfected RAW264.7 cells, IL-10 also induced up-regulation of B7-H4 expression (^##^P < 0.01). After miR-140 mimics were transfected into RAW264.7 cells, there was no significant difference between IL-10 treated group and no IL-10 treated group (^NS^P>0.05).

Hence, we confirm that miR-140 may negatively regulate B7-H4 expression in RAW264.7 cells and B7-H4 is a potential target gene of miR-140.

### 3.3 IL-10 inhibits the promoter activity of miR-140 in RAW264.7 cells

**Figure 3:**
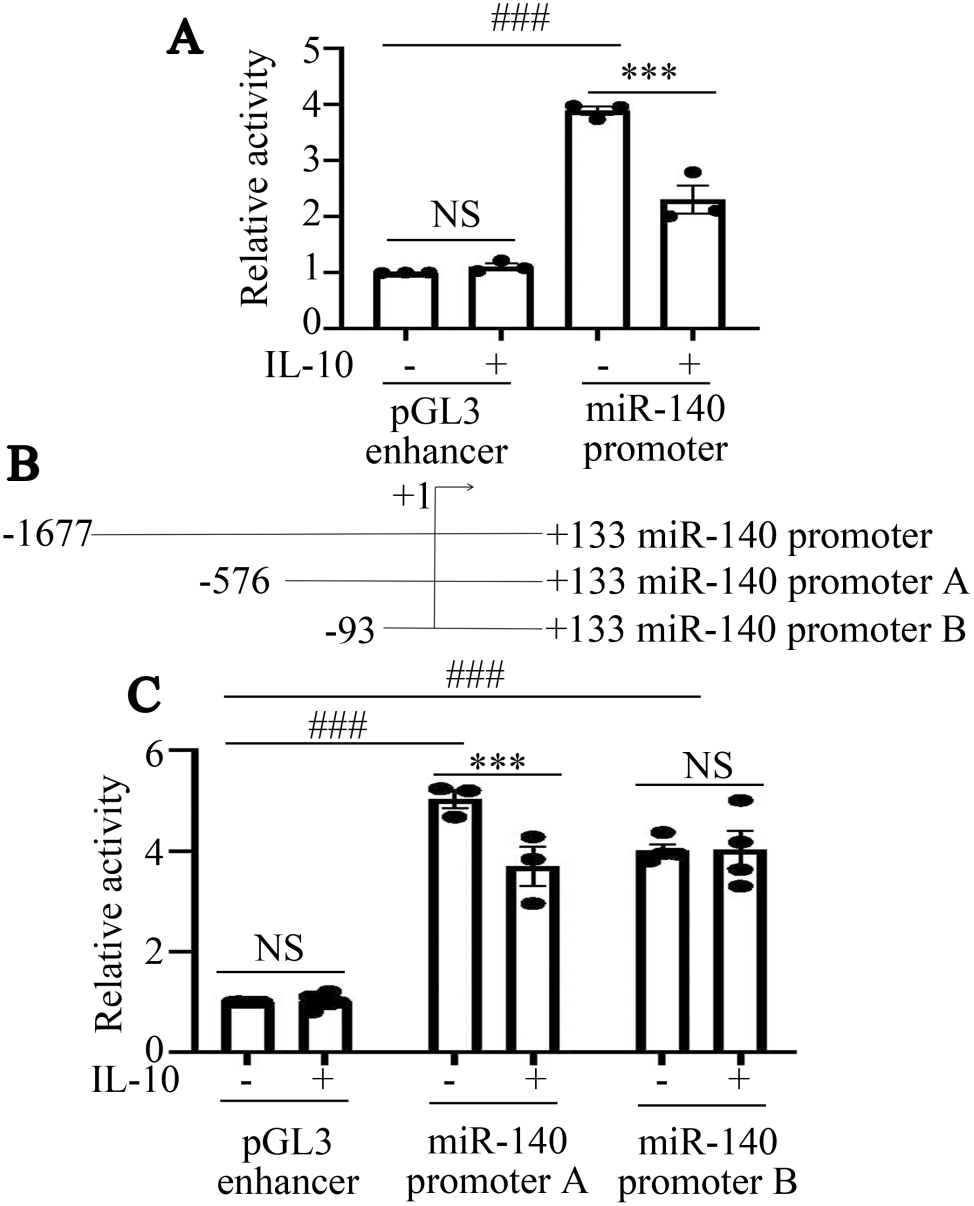
IL-10 inhibits the promoter activity of miR-140 in RAW264.7 cells. **(A)**, Fluorescence activity of miR-140 promoter following IL-10 treatment was assessed by dual luciferase reporter assay. *P<0.05; ^NS^P>0.05. One-way ANOVA statistical analysis (LSD) was used to analyze all the data among various groups. **(B)**, Schematic diagram of miR-140 promoter truncation fragments was shown. **(C)**, Fluorescence activities produced by miR-140 promoter A, miR-140 promoter B in RAW264.7 cells treated with or without IL-10 were assessed by dual luciferase reporter assay. ***P<0.001, ^###^P<0.001, ^NS^P>0.05. One-way ANOVA statistical analysis (LSD) was used to analyze all the data among various groups.

We further investigated the mechanism by which IL-10 regulated miR-140 expression in RAW264.7 cells. Firstly, a plasmid containing the whole miR-140 promoter sequence was constructed as miR-140 promoter. Luciferase activity in cells transfected with miR-140 promoter was higher than that in the pGL3 enhancer transfected cells (^###^P<0.001, **Figure 3A**). This result confirmed that the plasmid of miR-140 promoter was constructed successfully. IL-10 further suppressed luciferase activity produced by the miR-140 promoter, but had no effect on pGL3 enhancer (^***^P<0.001, ^NS^P>0.05, **Figure 3A**).

To narrow down the active region of the miR-140 promoter, we then constructed luciferase reporter plasmids containing three truncated fragments, miR-140 promoter A, miR-140 promoter B (**Figure 3B**). To further elucidate the potential mechanism by which IL-10 inhibits the activity of miR-140 promoter, we transfected pGL3 enhancer, miR-140 promoter A and miR-140 promoter B into RAW264.7 cells. Then the cells were stimulated with IL-10 (10 ng/mL) for 24 h. IL-10 significantly inhibited the luciferase activity in RAW264.7 cells transfected with miR-140 promoter A (^***^P<0.001, **Figure 3C**). In RAW264.7 cells transfected with miR-140 promoter B or pGL3 enhancer, there was no significant difference in luciferase activity between IL-10-treated and untreated groups (^NS^P>0.05, **Figure 3C**). Hence, according to dual luciferase reporter gene analysis, IL-10 may inhibit miR-140 promoter activity in RAW264.7 cells through transcription factors that binding to the − 576/- 94 bp region of the miR-140 promoter.

### 3.4 IL-10 downregulates miR-140 promoter activity in a STAT5 dependence manner

**Figure 4:**
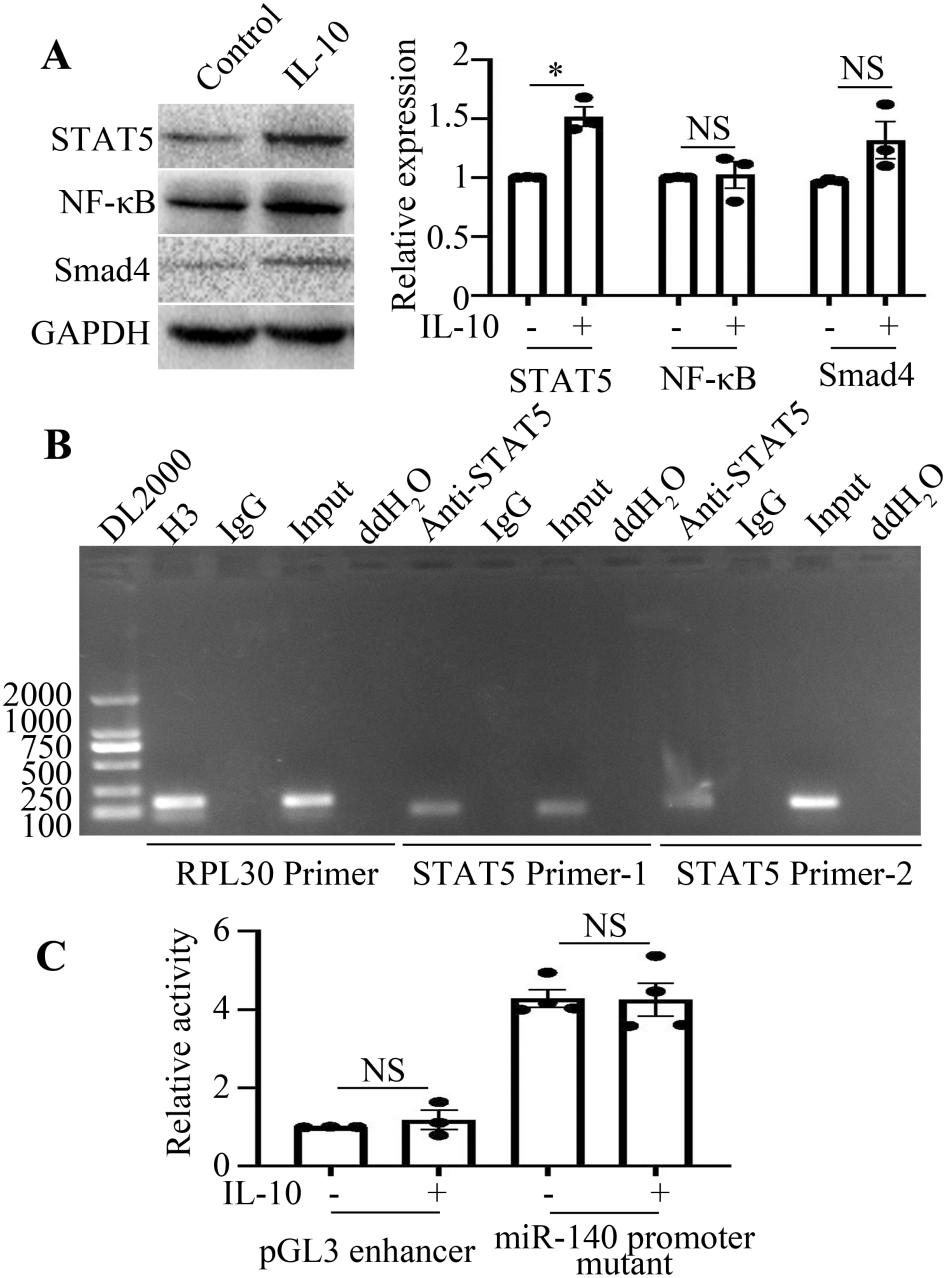
IL-10 downregulates miR-140 promoter activity in a STAT5 dependence manner. **(A)**, The expression levels of STAT5, Smad4 and NF-κB in the presence or absence of IL-10 treatment were detected by Western blot. The comparison between the two groups was conducted using Independent-samples T test. *P < 0.05, ^NS^P > 0.05, compared to each control group. **(B)**, Binding of STAT5 to miR-140 promoter was detected by ChIP assay. Both the RPL30 primer and H3 antibody were used as the positive control. **(C)**, Activities of pGL3 enhancer or miR-140 promoter mutant in IL-10 stimulated or unstimulated RAW264.7 cells were analyzed using the dual luciferase reporter enzyme assay. ^NS^P>0.05, compared to each control group. The comparison between the two groups was conducted using Independent-samples T test.

Transcription factors binding to the − 576/- 94 bp region of the miR-140 promoter were predicted and screened by the PROMO web online database. The prediction results suggested that transcription factors such as STAT5, Smad4 and NF-κB may act in this region. As the results shown in **Figure 4A**, IL-10 promoted STAT5 expression in RAW264.7 cells, whereas it had no effect on Smad4 and NF-κB expression. According to the results of ChIP analysis (**Figure 4B**), the transcription factor STAT5 can directly bind to the miR-140 promoter.

Next, we sought to explore the effect of STAT5 on the miR-140 promoter in IL-10-treated RAW264.7 cells. The targeted mutation was located at the STAT5 binding site within the − 456/− 446 bp region of the miR-140 promoter. After the plasmid miR-140 promoter mutant was transfected into RAW264.7 cells, IL-10 failed to suppress its activity in the group transfected with miR-140 promoter mutant (**Figure 4C**). This result and the results of **Figure 3** above demonstrated that IL-10 may suppress the activity of miR-140 promoter in a STAT5-dependent manner.

## 4 Discussion

Schistosomiasis is a zoonosis that seriously endangers human health. During schistosomiasis, Th1 inflammatory response is observed first in the hosts, accompanied with the production of tumor necrosis factor (TNF)-α and IL-12, et al (Chuah et al., 2014; Saad et al., 2022). Then with schistosome eggs laying, soluble egg antigens (SEA) released from schistosome eggs recruits a variety of immune cells, produces a Th2 immune inflammatory response and induces the formation of granulomas and liver fibrosis in hosts (Chuah et al., 2014; Saad et al., 2022). As innate immune cells, macrophages participate in the dynamic formation of schistosome egg granulomas and the development of liver fibrosis through differentiation into different phenotypes (Gordon, 2003; Zhu et al., 2023). On the one hand, macrophages play a role in the acute phase of schistosome infection, phagocytose necrotic tissue, and participate in regulating the process of liver fibrosis development. On the other hand, macrophages function as antigen-presenting cells (APCs) to activate T cells during immune responses (Gordon, 2003; Zhu et al., 2023). In general, the activation of T cells requires two signals. The first signal comes from the TCR recognizing the major histocompatibility complex (MHC) complex with the antigen peptide. The second signal is provided by co-stimulatory or co-inhibitory molecules expressed on the surface of APCs. In this process, B7-H4 expressed on various cells (including tumor cells and APCs) could be identified as a co-inhibitory molecule and may play an important role in the inhibition of TCR-mediated T cell proliferation (Ni and Dong, 2017). Since the immune negative regulation mechanism plays an important role in schistosomiasis (Zhou et al., 2016), the expression change and its potential role of B7-H4 in schistosomiasis are worth investigation. In our previous studies, we found that B7-H4 mRNA expression was upregulated in liver tissues from mice infected with *Schistosoma japonicum*. Its expression change trend was similar as IL-10 mRNA expression in mouse livers with schistosomiasis (Data not published). Meanwhile, IL-10 promoted up-regulation of B7-H4 expression in macrophages through JAK2/STAT3 signaling pathway (Data not published). In this study, we also found B7-H4 protein expression was obviously enhanced in mice infected with *Schistosoma japonicum*.

miRNAs are non-coding RNAs *in vivo* that are widespread in a variety of organisms and can regulate many physiopathological processes (Gao et al., 2022). miRNAs also participate in the development of schistosomiasis (Hong et al., 2017). For example, elevated miR-146a/b plays a protective role in schistosomiasis by inhibiting IFN-γ induced macrophage differentiation into M1 cells through targeting STAT1 during the development of Schistosomiasis japonica (He et al., 2016). In our studies, we found that B7-H4 may be a potential target gene of miR-140 in IL-10 treated RAW264.7 cells. Hence, miR-140 may regulate B7-H4 expression and play an important role in immunoregulation in Schistosomiasis japonica. It has been found that miR-140 negatively regulates its expression by targeting CAPN1 in liver tissue AML12 cells, thereby protecting liver ischemia-reperfusion (Yu et al., 2021). Studies also confirmed that loss of TUG1 could alleviate LPS-induced hepatocyte inflammation and injury by modulating the miR-140/TNF axis (Liu et al., 2020). In this study, we observed that miR-140 expression was decreased in IL-10-treated RAW264.7 cells. We further confirmed that miR-140 could bind to the B7-H4 3’UTR region and ultimately promote B7-H4 expression in RAW264.7 cells.

Transcription factors play messenger roles in the regulation of gene expression, either promoting miRNA transcription, or inhibiting miRNA transcription. NRF2 may directly bind to the promoter of miR-140 in human lung fibroblasts (HLFs) (Duru et al., 2015). And nuclear translocation of NRF2 may contribute to the enhanced expression of miR-140 upon ionizing radiation (IR) treatment. In breast cancer, Ying Yang 1 (YY1), which is a kind of transcription factor/repressor also directly binds to the miR-140 promoter and promotes the expression of miR-140 (Lu et al., 2020). In our previous researches on *Schistosoma japonicum*, we also confirmed that the transcription factor STAT5 could bind to the promoter of miR-155 and suppress the activity of the miR-155 promoter (Zhu et al., 2018). In this study, we find that the transcription factor STAT5 directly binds to miR-140 transcription at positions from − 456/− 446 bp region of the miR-140 promoter and STAT5 may inhibit miR-140 expression in RAW264.7 cells.

**Figure 5:**
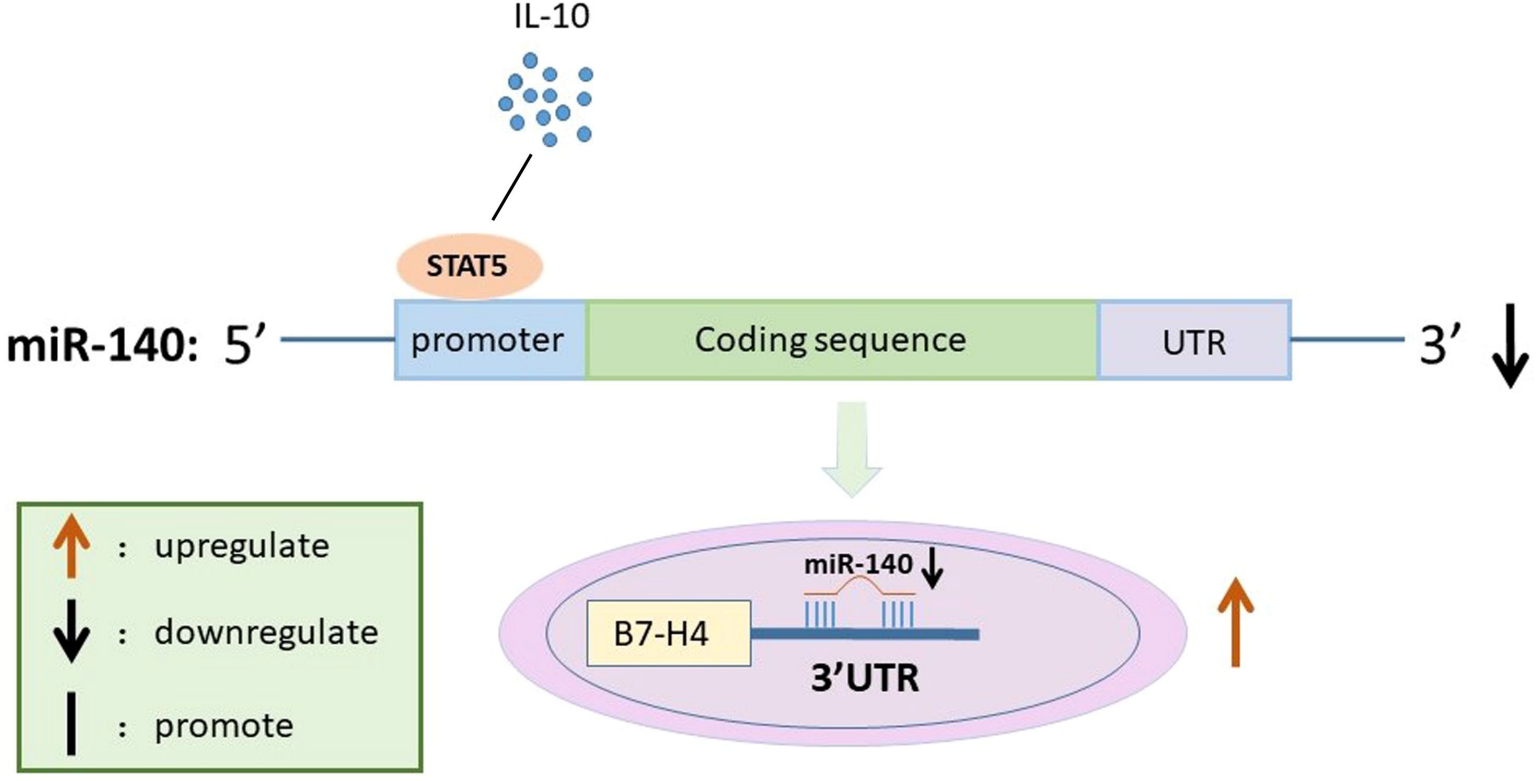
Schematic diagram for the mechanism by which miR-140 promotes B7-H4 expression in IL-10-treated RAW264.7 cells. IL-10 suppresses miR-140 promoter activity by affecting STAT5 expression. miR-140 then directly targets to the 3’UTR of B7-H4, which in turn leads to up-regulation of B7-H4 expression in IL-10 treated RAW264.7 cells.

In conclusion, IL-10 promotes STAT5 expression and inhibit miR-140 expression, thereby inducing B7-H4 expression in RAW264.7 cells (**Figure 5**). Although RAW264.7 cells we used in this study are widely used for macrophage polarization and macrophage biology studying, some difference between primary macrophages and cell lines exist (Qie et al., 2022). Moreover, emerging evidence highlights diverse populations of tissue-resident immune cells (including Kupffer cells and liver-resident NK cells, et al.) dynamically interact with hepatocytes and hepatic stellate cells (Narmada et al., 2024; Weiskirchen and Tacke, 2014). This intricate cellular network establishes specialized immune surveillance mechanisms that are critical for maintaining hepatic homeostasis. To comprehensively characterize B7-H4’s pathophysiological roles, future investigations are warranted to elucidate the precise immunoregulatory mechanisms through which B7-H4 modulates the complex interplay between parenchymal and non-parenchymal cell populations in both physiological and pathological states.

## Data Availability

The original contributions presented in the study are included in the article/**Supplementary Material**. Further inquiries can be directed to the corresponding author. “IL-10/STAT5 Axis Suppresses miR-140 to Upregulate B7-H4 Expression in RAW264.7 cells”, Mendeley Data, V2, doi: 10.17632/rpd7zntnfc.2.
