# Supplementary material for: IL-10/STAT5 axis suppresses miR-140 to upregulate B7-H4 expression in RAW264.7 cells
Source: Front Cell Infect Microbiol. 2025 Aug 15;15:1613297. doi: 10.3389/fcimb.2025.1613297 (PMC12394516; doi:10.3389/fcimb.2025.1613297)
Supplement: Supplementary file 1 [file Table1.docx]

Supplemental Table 1. Primers for plasmids construction

| Plasmids | Primers | 5’ → 3’ |
| --- | --- | --- |
| B7-H4 3'UTR wild | forward | CCGCTCGAGGTCCGTCTTCTCATTCC |
|  | reverse | AGCTTTGTTTAAACTGTAACTGTTCGTCCATGG |
| B7-H4 3'UTR mutant | forward | ACTCCAAGTGCCTTTTTTATCAAGGTCAGG |
|  | reverse | CCTGACCTTGATAAAAAAGGCACTTGGAGT |
| miR-140 promoter | forward | CGGGGTACCATTGCTGTCGGGTTGGTCT |
|  | reverse | CCCAAGCTTGCTGGGCTTTGTGGCTGT |
| miR-140 promoter A | forward | CGGGGTACCACAAATCAGGTCCAGGCCT |
|  | reverse | CCCAAGCTTGCTGGGCTTTGTGGCTGT |
| miR-140 promoter B | forward | CGGGGTACCGTTCTGTTTTCGGTGTGACC |
|  | reverse | CCCAAGCTTGCTGGGCTTTGTGGCTGT |
| miR-140 promoter mutant | forward | CAGAGCAGGTTAAGTCAGAAAAAAAAAAACACATCTGGTATGTC |
|  | reverse | GACATACCAGATGTGTTTTTTTTTTTCTGACTTAACCTGCTCTG |

Supplemental Table 2. Primers for ChIP

| Name | Primers | 5’ → 3’ |
| --- | --- | --- |
| STAT5 Primer-1 | forward | CCCCTGGCTTTCCTTCTA |
|  | reverse | AGGAGTGGCAATGCTTAGAC |
| STAT5 Primer-2 | forward | GTGTCCCGTAAAATCAGCCC |
|  | reverse | ACTGGTACACAGGACATGCAGC |

Supplemental Table 3. Antibodies used in this study

|  | Name | Company | Catalog number | Dilution |
| --- | --- | --- | --- | --- |
| Primary  antibody | Rabbit monoclonal antibody [EPR23665-20] to B7H4 | Abcam | ab252438 | 1:1000 |
|  | Rabbit polyclonal antibody to STAT5 | Cell Signaling Technology | 9363 | 1:1000 for WB  1:50 for ChIP |
|  | Rabbit polyclonal antibody to NF-κB p65 (C-20) | Santa Cruz Biotechnology | sc-372 | 1:200 |
|  | Mouse monoclonal antibody to Smad4 (B-8) | Santa Cruz Biotechnology | sc-7966 | 1:200 |
|  | Mouse monoclonal antibody to GAPDH | Proteintech | 60004-1-Ig | 1:50000 |
| Secondary antibody | Goat anti-mouse IgG-HRP | Santa Cruz Biotechnology | sc-2005 | 1:4000 |
|  | Goat anti-rabbit IgG-HRP | Biosharp | BL003A | 1:50000 |

Supplemental Table 4. Primers for microRNAs

| Name | Primers | 5’ → 3’ |
| --- | --- | --- |
| mmu-miR-140 | CAGTGGTTTTACCCTATGGTAG | |
| mmu-miR-155 | AATGACACGATCACTCCCGTTGA | |
| mmu-miR-96 | TTTGGCACTAGCACATTTTTGCT | |
| mmu-miR-107 | AGCAGCATTGTACAGGGCTATCA | |
| mmu-miR-425 | AATGACACGATCACTCCCGTTGA | |
| mmu-miR-181c | AACATTCAACCTGTCGGTGAGT | |
| mmu-miR-23a | ATCACATTGCCAGGGATTTCC | |

Supplemental Table 5. PCR amplification conditions

| Stage | Temperature | Time | Number of cycles |
| --- | --- | --- | --- |
| Holding stage | 95°C | 30s | 1 |
| Cycling stage | 95°C | 5s | 40 |
|  | 62°C | 30s |  |
|  | 72°C | 30s |  |
| Melt curve stage | 95°C | 15s | 1 |
|  | 62°C | 30s |  |
|  | 95°C | 15s |  |
